# Letter to the editor—Mortality rate of acute kidney injury in SARS, MERS, and COVID-19 infection: a systematic review and meta-analysis

**DOI:** 10.1186/s13054-020-03239-0

**Published:** 2020-09-11

**Authors:** Joel Swai

**Affiliations:** Department of Nephrology, Benjamin Mkapa Hospital, P.O Box 11088, 41218 Dodoma City, Tanzania

Dear Editor,

I have read with interest the published article entitled “Mortality rate of acute kidney injury in SARS, MERS, and COVID-19 infection: a systematic review and meta-analysis” by Chen et al. [1]. The article is well written, and I have three concerns as explained below.

Firstly, the mortality rate for COVID-19 patients with AKI is different in the text (i.e., 76.5%; 95% CI 61.0–89.0) from one reported in the authors’ Figure 1 (i.e., 78.0%; 95% CI 63.0–90.0). The authors might need to clarify this discrepancy.

Secondly, the authors mistakenly made a duplicate entry of the study by Chen et al. (2020) in the COVID-19 forest plot. This mistake resulted in a pooled AKI mortality rate of 78.0% (CI 63.0–90.0), *I*^2^ = 97.1%, *P* < 0.0001, instead of 53.99% (CI 52.34–55.65), *I*^2^ = 98.4%, *P* < 0.0001, had the authors sorted the duplicate-entry problem.

Thirdly, the authors concluded the mortality rate for COVID-19 patients with AKI from an otherwise a high heterogeneity of *I*^2^ = 97.1%, *P* < 0.0001. This strongly impacts the reliability of the conclusion drawn [2].

I, on the other hand, reanalyzed authors’ data and performed sensitivity analysis according to the Cochrane Library recommendation [3]. I excluded six peculiar studies from the analysis. Alberici et al. and Banerjee et al. involved kidney transplant patients, unlike the rest of the studies. Wang et al. utilized intensive care unit patients, unlike other studies. Moreover, Alberici et al., Banerjee et al., Hirsch et al., Suwanwongse et al., and Richardson et al. included racially diverse participants. Different races have different COVID-19 mortalities [4, 5]. The nine remaining studies represented all-Asian Chinese hospitalized patients with COVID-19 and AKI. The newly obtained mortality rate for COVID-19 patients with AKI was 94.90% (CI 91.47–98.34), with non-statistically significant heterogeneity, *I*^2^ = 7.4%, *P* < 0.375, see Fig. 1. Sensitivity analysis could not be conducted in MERS and SARS outcomes because of an insufficient number of studies.

**Fig. 1:**
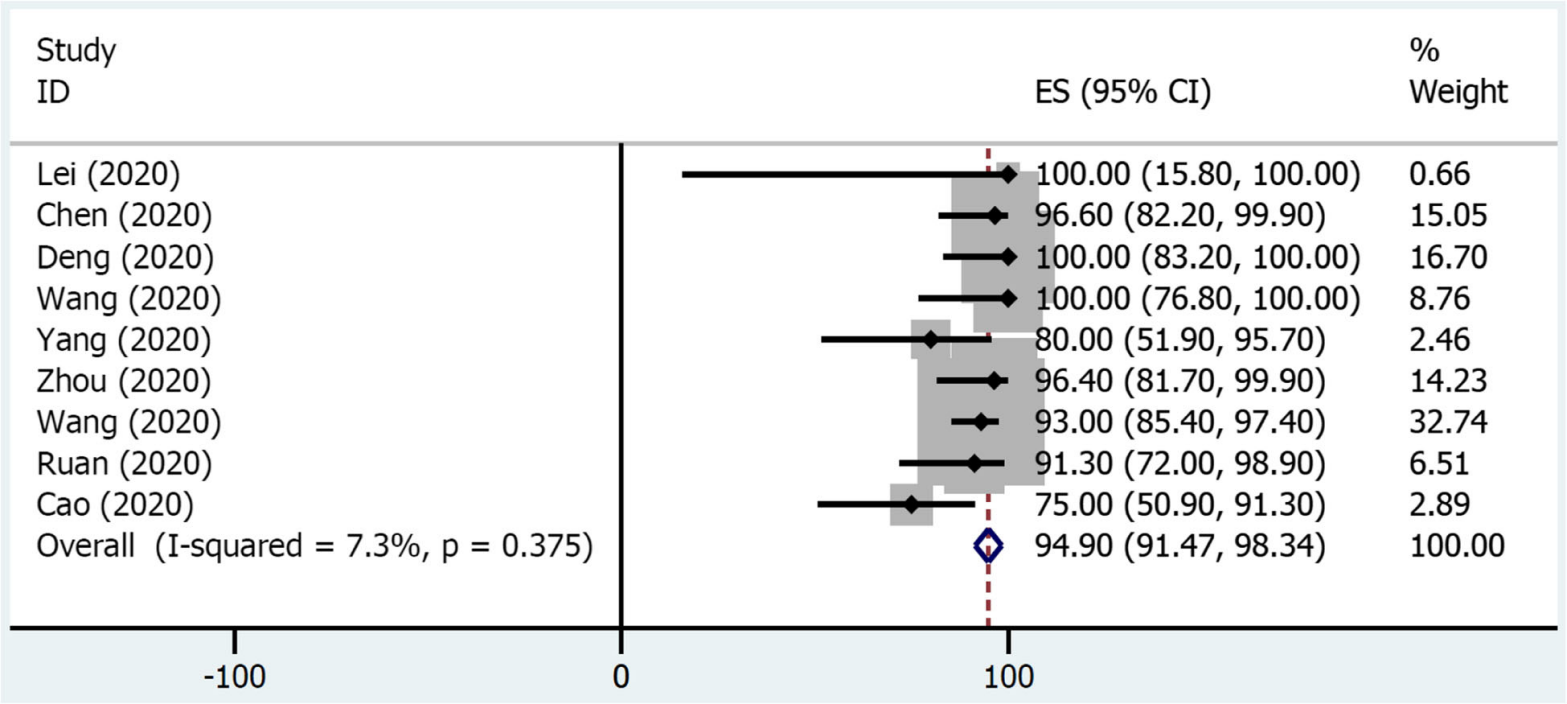
A forest plot of AKI mortality in coronavirus infections from included studies: COVID-19

## Data Availability

Not applicable.
