# Supplementary material for: Letter to the editor—Mortality rate of acute kidney injury in SARS, MERS, and COVID-19 infection: a systematic review and meta-analysis
Source: Crit Care. 2020 Sep 11;24:555. doi: 10.1186/s13054-020-03239-0 (PMC7485206; doi:10.1186/s13054-020-03239-0)
Supplement: Related texts

# Reply: Authors’ response to “Joel Swai. Letter to the editor—Mortality rate of acute kidney injury in SARS, MERS, and COVID-19 infection: a systematic review and meta-analysis”

**Authors:** Yih-Ting Chen, Shih-Chieh Shao, Yung-Chang Chen

We thank Dr. Joel Swai for the interest in our research letter. As the author pointed out, the reported results of mortality rate for coronavirus disease 2019 (COVID-19) patients with acute kidney injury (AKI) is different from the text and Figure 1c in the original publication of our article [1]. The problem is that we mistakenly made a duplicate entry of the Chen et al. (2020) study while performing the COVID-19 forest plot. We have noticed this critical issue and sent the correct proof before the article publication; unfortunately, this mistake had not been accurately revised by the production team. We sincerely regret the inaccuracy may cause any inconvenience to the readers. However, there are no changes to the interpretation of the results, conclusions, and applications of our article. In details:

1. There was no evidence of statistical heterogeneity among studies reporting AKI mortality in SARS (*I*^2^ = 0.0%, *P* = 0.589) and MERS (*I*^2^ = 0.0%, *P* = 0.758), but there was for COVID-19 infection (*I*^2^ = 97.0%, *P* < 0.0001).
2. Figure 1c:

In addition, our research letter aimed to overview the AKI mortality in patients with different coronaviruses, but the clinical heterogeneity between studies should be also noted. One of the important factors may be the racial difference between studies as Dr. Joel Swai noted. Even if the recent study from Fisher et al. indicated the races are not associated with mortality in COVID-19 patients developing AKI [6], future large meta-analyses may be suggested to explore the clinical impacts from different races in AKI mortality in COVID-19 patients.
